# Usefulness of synthetic MRI for differentiation of IDH-mutant diffuse gliomas and its comparison with the T2-FLAIR mismatch sign

**DOI:** 10.1007/s11060-024-04794-0

**Published:** 2024-08-12

**Authors:** Shumpei Onishi, Fumiyuki Yamasaki, Yuji Akiyama, Daisuke Kawahara, Vishwa Jeet Amatya, Ushio Yonezawa, Akira Taguchi, Iori Ozono, Novita Ikbar Khairunnisa, Yukio Takeshima, Nobutaka Horie

**Affiliations:** 1 Department of Neurosurgery, Graduate School of Biomedical and Health Sciences, Hiroshima University, 1-2-3 Kasumi, Minami-ku, Hiroshima-city, Hiroshima 734-8551 Japan; 2 Department of Clinical Radiology, Hiroshima University Hospital, Hiroshima, Japan; 3 Department of Radiation Oncology, Graduate School of Biomedical Health Sciences, Hiroshima University, Hiroshima, Japan; 4 Department of Pathology, Graduate School of Biomedical and Health Sciences, Hiroshima University, Hiroshima, Japan

**Keywords:** T2-FLAIR mismatch sign, Diffuse glioma, Astrocytoma, IDH-mutant, Synthetic MRI

## Abstract

**Introduction:**

The T2-FLAIR mismatch sign is a characteristic imaging biomarker for astrocytoma, isocitrate dehydrogenase (IDH)-mutant. However, investigators have provided varying interpretations of the positivity/negativity of this sign given for individual cases the nature of qualitative visual assessment. Moreover, MR sequence parameters also influence the appearance of the T2-FLAIR mismatch sign. To resolve these issues, we used synthetic MR technique to quantitatively evaluate and differentiate astrocytoma from oligodendroglioma.

**Methods:**

This study included 20 patients with newly diagnosed non-enhanced IDH-mutant diffuse glioma who underwent preoperative synthetic MRI using the Quantification of Relaxation Times and Proton Density by Multiecho acquisition of a saturation-recovery using Turbo spin-Echo Readout (QRAPMASTER) sequence at our institution. Two independent reviewers evaluated preoperative conventional MR images to determine the presence or absence of the T2-FLAIR mismatch sign. Synthetic MRI was used to measure T1, T2 and proton density (PD) values in the tumor lesion. Receiver operating characteristic (ROC) curve analysis was performed to evaluate the diagnostic performance.

**Results:**

The pathological diagnoses included astrocytoma, IDH-mutant (*n* = 12) and oligodendroglioma, IDH-mutant and 1p/19q-codeleted (*n* = 8). The sensitivity and specificity of T2-FLAIR mismatch sign for astrocytoma were 66.7% and 100% [area under the ROC curve (AUC) = 0.833], respectively. Astrocytoma had significantly higher T1, T2, and PD values than did oligodendroglioma (*p* < 0.0001, < 0.0001, and 0.0154, respectively). A cutoff lesion T1 value of 1580 ms completely differentiated astrocytoma from oligodendroglioma (AUC = 1.00).

**Conclusion:**

Quantitative evaluation of non-enhanced IDH-mutant diffuse glioma using synthetic MRI allowed for better differentiation between astrocytoma and oligodendroglioma than did conventional T2-FLAIR mismatch sign. Measurement of T1 and T2 value by synthetic MRI could improve the differentiation of IDH-mutant diffuse gliomas.

**Supplementary Information:**

The online version contains supplementary material available at 10.1007/s11060-024-04794-0.

## Introduction

Isocitrate dehydrogenase (IDH)-mutant diffuse gliomas can be classified into astrocytoma and oligodendroglioma based on the status of chromosomes 1p and 19q [1, 2]. Classification based on molecular phenotype is more effective than histopathological diagnosis for estimating prognosis, and preoperative differential diagnosis of these two entities helps decision of therapeutic strategies, including extent of surgical resection. Patients with astrocytoma, IDH-mutant need to undergo more aggressive surgical resection considering that correlation between greater extent of resection and survival as suggested by previous studies [3–5]. Given that oligodendroglioma, IDH-mutant and 1p/19q-codeleted is sensitive to chemo- and radiotherapy [6, 7], preoperative identification of the molecular subtype of diffuse gliomas is critical.

Studies have shown that the T2-fluid-attenuated inversion recovery (FLAIR) mismatch sign is a highly specific imaging biomarker for astrocytoma, IDH-mutant [8–10]. The T2-FLAIR mismatch sign is a homogeneously or near homogeneously hyperintense signal on T2-weighted imaging (T2WI) and a relatively homogeneously or near homogeneously hypointense signal on FLAIR images except for a circumjacent hyperintense rim [8]. However, investigators occasionally provide varying interpretations of the sign due to the nature of qualitative visual assessment. Moreover, the MR sequence parameters also influence the appearance of the T2-FLAIR mismatch sign, particularly the inversion time (TI) for acquiring FLAIR [11]. Although the previous cohort study showed that the FLAIR scanned with TI less than 2,400 ms in 3 Tesla (T) magnetic resonance (MR) machine improved the detectability of T2-FLAIR mismatch sign, not all the astrocytoma, IDH-mutant present T2-FLAIR mismatch sign [12].

The conventional inversion recovery (IR) or multiple spin echo (MSE) sequence can obtain the quantitative T1 and T2 value with an additional more than 20 min [13], while the development of synthetic MRI has enabled the production of image contrasts with variable T1 and T2 weighting from a single series of a few minutes scan data and quantification of T1, T2, and proton density value [14]. The imaging quality of the synthetic MR imaging were noninferior compared with the conventional MR imaging, but synthetic T2 FLAIR reconstructions was inferior to conventional FLAIR image due to the profound artifacts [15]. Therefore, the evaluation of T2-FLAIR mismatch sign with synthetic MR imaging may have the difficulty of interpretation. As for the quantitative analysis, conventional MR required the calculation of relative signal intensity. Meanwhile, quantitative measurement of T1, T2, and proton density values obtained from synthetic MRI allowed direct comparison of subjects with an objective manner with high reliability [16, 17]. In the previous study, astrocytoma could be differentiated from oligodendroglioma by using quantitative evaluation of T2 value with synthetic MRI [18]. Furthermore, pre- and post- contrast T1 values obtained from synthetic MRI have also been used to predict IDH status in diffuse gliomas [19].

This study therefore aimed to utilize and validate quantitative evaluation of T1 and T2 values obtained from synthetic MRI in order to differentiate astrocytoma from oligodendroglioma. We hypothesized that the synthetic MRI and quantitative evaluation of these parameters would improve the diagnostic accuracy of non-enhanced IDH-mutant diffuse gliomas.

## Methods

### Patients

This retrospective study was approved by the Ethical Committee for Epidemiology of Hiroshima University (E2022-0038). Due to the retrospective nature of the study, the Ethical Committee for Epidemiology of Hiroshima University waived the need of obtaining informed consent. All methods were performed in accordance with the relevant guidelines and regulations. We included 20 adult patients newly diagnosed with non-enhanced IDH-mutant diffuse glioma who underwent preoperative synthetic MRI using the Quantification of Relaxation Times and Proton Density by Multiecho acquisition of a saturation-recovery using Turbo spin-Echo Readout (QRAPMASTER) sequence at our institute from January 2019 to April 2024.

### Histopathological diagnosis and molecular signature analysis

Tumor specimens after surgical resection were fixed in 10% phosphate-buffered formalin and embedded in paraffin blocks. Representative specimens were then stained with hematoxylin–eosin reagents for standard histological diagnosis.

The tumors were diagnosed based on the World Health Organization Classification of Tumors of the Central Nervous System updated in 2021 [2]. Immunohistochemical (IHC) staining for all antibodies was performed in an automated immunostainer (BenchMark GX; Ventana). IHC staining for IDH1-R132H and α-thalassemia X-linked intellectual disability (ATRX) was performed. Fluorescence in situ hybridization analysis for 1p/19q was performed as described previously [20]. Astrocytoma, IDH-mutant with histological features (microvascular proliferation or necrosis) or homozygous deletion (HD) of CDKN2A/B were classified as CNS WHO grade 4. Instead of the direct confirmation of CDKN2A status, loss of methylthioadenosine phosphorylase (MTAP) of immunohistochemiecal staining was used as a surrogate marker for CDKN2A HD [21, 22]. Immunohistochemical staining for MTAP was performed with anti-MTAP mouse monoclonal antibody, clone 2G4 (1:100, Abnova).

### Conventional MR/computed tomography (CT) image acquisition and evaluation

Conventional MR scans were acquired using a 3-T MR scanner (Ingenia CX 3.0 T; Philips Healthcare, Best, Netherlands) with a 32-channel head coil. The conventional MR scans include T2-weighted image (WI) (TR: 3,000 ms; TE: 100 ms; echo train length: 15; FOV: 220 mm; matrix size: 512 × 387 (reconstruction 640 × 640); number of excitations (NEX): 2; section thickness: 5 mm; intersection gap: 1.0 mm; 24 slices; and 1 acquisition; scan time: 2 min and 36 s) and FLAIR image (TR: 10,000 ms; TE: 130 ms; inversion recovery time: 2600 ms; FOV: 220 mm, matrix size: 288 × 230 (reconstruction 512 × 512); NEX: 1; section thickness: 5 mm; intersection gap: 1.0 mm; 24 slices, and 3 acquisitions; scan time: 3 min and 0s). Investigators evaluated T1WI, T2WI, FLAIR, DWI, T2*WI and post-enhanced T1WI sequences of each patient. Details of MR sequence parameters were described previously [23]. 

The T2-FLAIR mismatch was assessed using an approach similar to that described previously [24]. The sign represented (1) a complete/near-complete hyperintense signal on T2WI and (2) relatively hypointense signal on FLAIR except for a hyperintense peripheral rim [24]. Moreover, we used additional imaging features for the accurate identification of the T2-FLAIR mismatch sign as previously described [9]. (1) Necrotic cavities do not represent the T2-FLAIR mismatch sign, whereas small cysts do not sufficiently satisfy the criteria for T2-FLAIR mismatch. (2) The T2-FLAIR mismatch sign is typically accompanied by little or no contrast enhancement as the previously published criteria. (3) Furthermore, the degree of FLAIR signal suppression may be inhomogeneous within the tumor.

CT was performed on a 320-detector CT scanner (Aquilion ONE; Canon Medical Systems). Intratumoral hemorrhage was defined as hypo-intensity on T2*WI excluding calcification assessed using CT images.

Two independent investigators (S.O. and F.Y.) independently assessed the presence or absence of the T2-FLAIR mismatch sign, contrast enhancement, cystic component, calcification, and intratumoral hemorrhage.

### Synthetic MR image acquisition and evaluation

Synthetic MRI using the QRAPMASTER sequence with two echo times (TE; 13 and 100 ms) and four delayed time was performed using the same 3-T MR scanner (Ingenia CX 3.0 T). The parameters included TR: 4,000 ms; echo train length, 10; FA: 90°; sensitivity encoding factor, 3; FOV 220 mm; matrix size: 256 × 153 (reconstruction 512 × 512); NEX: 1; section thickness: 5 mm; intersection gap: 1.0 mm; 24 slices, and scan time 2 min and 16 s. Quantification maps were generated and quantitative data were acquired using SyMRI^®^ (software version 11.0., Synthetic MR AB, Linköping, Sweden).

A single maximum section of each tumor was used for region of interest (ROI) analysis. ROIs were drawn to measure T1, T2, and PD values in the tumor lesion. The mean values of each parameter in the ROIs were obtained using SyMRI^®^. ROIs were placed avoiding cystic component, calcification, and intratumoral hemorrhage.

### Statistical analysis

Fisher’s exact test was used to compare the patients’ characteristics. Interrater agreement in the T2-FLAIR mismatch sign was evaluated using the Kappa statistic (κ = 0–0.40, 0.41–0.60, 0.61–0.80, and 0.81–1.00 indicating poor, moderate, good, and excellent agreement, respectively). The T2-FLAIR mismatch sign on conventional MRI was assessed between astrocytoma, IDH-mutant and oligdendroglioma, IDH-mutant and 1p/19q codeleted. T1, T2 and PD values were also analyzed between these group using the Mann–Whitney *U* test. The performance of each parameter and T2-FLAIR mismatch sign for the diagnosis of astrocytoma, IDH-mutant was evaluated using ROC curve analysis. Statistical analyses were performed using JMP pro version 17.0 (SAS institute, Cary, NC, USA) and GraphPad Prism version 7.00 for Mac (GraphPad Software, San Diego California, USA), with *P* values < 0.05 indicating statistical significance.

## Results

### Clinical and molecular pathological features

A total of 20 patients (9 females and 11 males; median age 39 years; range, 19–69 years) were included in this study. All cases showed little or no contrast enhancement according to the previously described criteria [9]. Among the included patients, 12 were pathologically diagnosed with astrocytoma, IDH-mutant (9 cases with grade 2, 2 case with grade 3, and 1 case of grade 4). One case of astrocytoma, IDH-mutant pathologically showed microvascular proliferation and necrosis, and was classified as grade 4. None of the astrocytoma, IDH-mutant showed loss of MTAP immunohistochemical staining. Whereas 8 were pathologically diagnosed with oligodendroglioma, IDH-mutant and 1p/19q-codeleted (7 cases with grade 2 and 1 cases with grade 3). Astrocytoma, IDH-mutant were located in the frontal lobe (*n* = 8), insula (*n* = 3), temporal lobe (*n* = 1), and parietal lobe (*n* = 1). Oligodendroglioma, IDH-mutant and 1p/19q-codeleted were located in the frontal lobe (*n* = 7) and parietal lobes (*n* = 1).

### T2-FLAIR mismatch sign and conventional MRI/CT characteristics

Among the included cases, 8 cases exhibited the positive T2-FLAIR mismatch sign. The interrater agreement for the positive T2-FLAIR mismatch sign was excellent (κ = 1.00). The pathological diagnosis of all T2-FLAIR mismatch positive cases was astrocytoma, IDH-mutant. The sensitivity and specificity of the T2-FLAIR mismatch sign for astrocytoma were 66.7% and 100% [area under the ROC curve (AUC) = 0.833]. None of the cases showed contrast enhancement, consistent with the definition of the T2-FLAIR mismatch sign. One case with oligodendroglioma exhibited iso-intensity on T1WI, whereas another case with oligodendroglioma and all cases with astrocytoma showed low intensity on T1WI. Calcification was more likely to be observed in oligodendroglioma than in astrocytoma (*p* = 0.0194, Fisher’s exact test). Intratumoral hemorrhage and cyst component also showed no significant difference in their ability to differentiate astrocytoma from oligodendroglioma (*p* = 1.000, 0.400, respectively. Fisher’s exact test).

A summary of the included cases is presented shown in Table 1.

**Table 1:** Characteristics of diffuse glioma patients

	Astrocytoma, IDH-mutant	Oligodendroglioma, IDH-mutant and 1p/19q codeleted	*p* value	
No. of patients	12	8		
Median age (range) (years)	37 (19–65)	40.5 (34–69)	0.1293	
Gender (M/F)	7 / 5	4 / 4	1.0000	
WHO grade (2/3/4)	9 / 2 / 1	7 / 1		
T2-FLAIR mismatch sign	8 (66.7%)	0 (0%)	0.0047	*
Cystic component	0 (0%)	1 (12.5%)	0.4000	
Calcification	2 (16.7%)	6 (75.0%)	0.0194	*
Intratumoral microbleeding	0 (0%)	0 (0%)	1.0000	

### Synthetic MRI parameters and diffuse glioma diagnosis

**Fig. 1:**
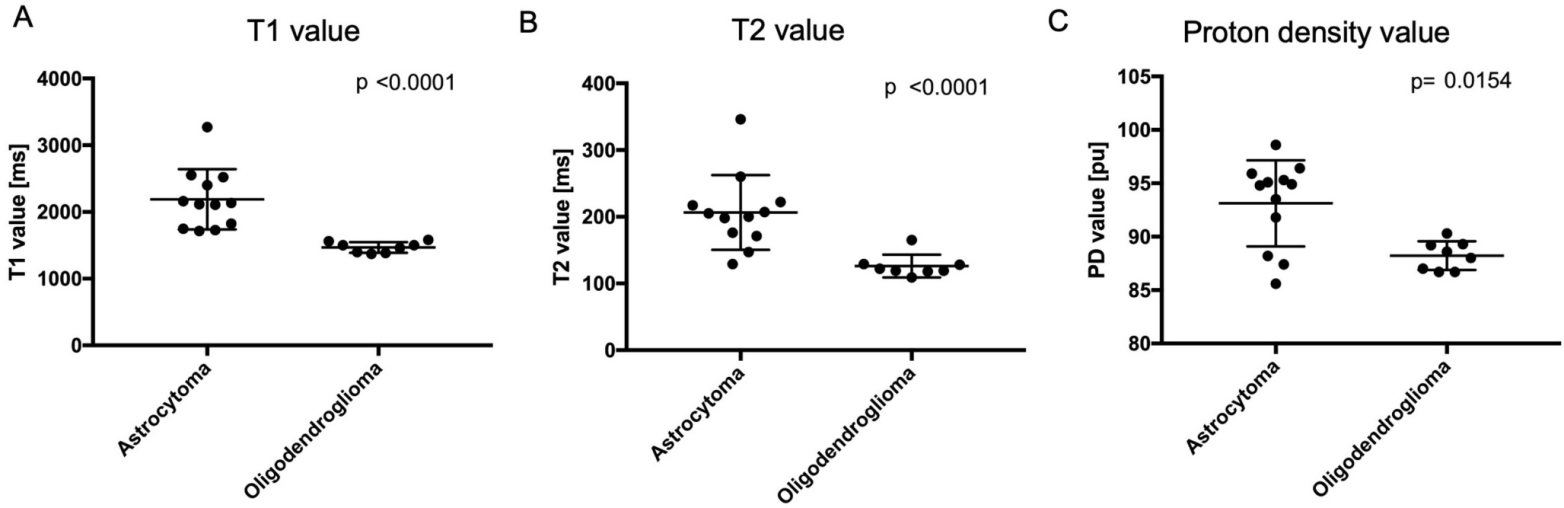
The scatter plots with bar comparing the synthetic magnetic resonance imaging parameters of astrocytoma and oligodendroglioma. (**A**) T1 value, (**B**) T2 value, and (**C**) proton density value in the tumor lesion

The mean T1, T2, and PD values and their standard deviations of tumor lesion are shown in Table 2; Fig. 1.

**Table 2:** Comparison of synthetic MRI parameters of astrocytoma, IDH-mutant, and oligodendroglioma, IDH-mutant and 1p/19q codeleted

	Astrocytoma, IDH-mutant	Oligodendroglioma, IDH-mutant, 1p/19q codeleted	*p* value	
No. of patients	12	8		
T1 value [ms]	2189.8 ± 450.8	1469.5 ± 79.8	< 0.0001	*
T2 value [ms]	206.5 ± 56.0	126.1 ± 16.9	< 0.0001	*
Proton density value [pu]	93.1 ± 4.0	88.2 ± 1.3	0.0154	*

The T1, T2, and PD values of the tumor lesion were significantly higher in astrocytoma cases (*p* < 0.0001, 0.0001, and 0.0154, Mann–Whitney *U* test, respectively) than in oligodendroglioma cases (Fig. 1A, B, C). A T1 cutoff value of 1580 ms (100% sensitivity, 100% specificity, and AUC = 1.00) was able to completely differentiated astrocytoma from oligodendroglioma. A T2 cutoff value of 171 ms was able to predict astrocytoma with 83.3% sensitivity and 100% specificity (AUC = 0.974) and PD cutoff value of 91.8 pu was able to predict astrocytoma with 75.0% sensitivity and 100% specificity (AUC = 0.823).

**Fig. 2:**
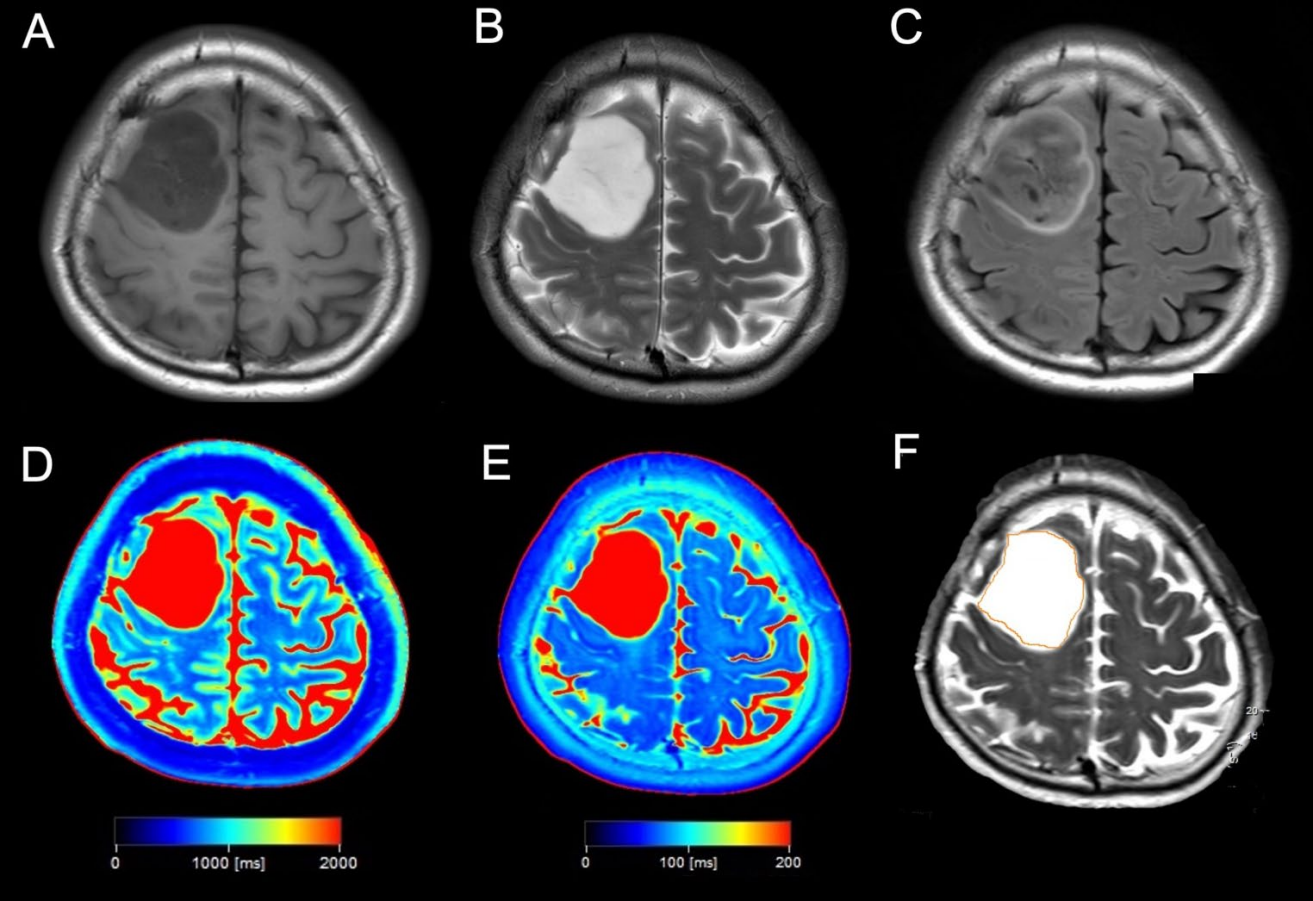
A case of a 42-year-old man with astrocytoma, IDH-mutant, grade 2. (**A**) T1-weighted imaging shows a homogeneous hypointense tumor in the right frontal lobe. (**B**) T2-weighted imaging shows a homogeneous hyperintense tumor in the right frontal lobe. (**C**) Fluid-attenuated inversion recovery showing signal suppression in the central tumor lesion with a hyperintensity peripheral rim, indicating a positive T2-FLAIR mismatch sign. (**D**, **E**) T1 (**D**) and T2 (**E**) maps showing prolonged T1 and T2 values in the tumor lesion. (**F**) The ROI was drawn in tumor lesion.

**Fig. 3:**
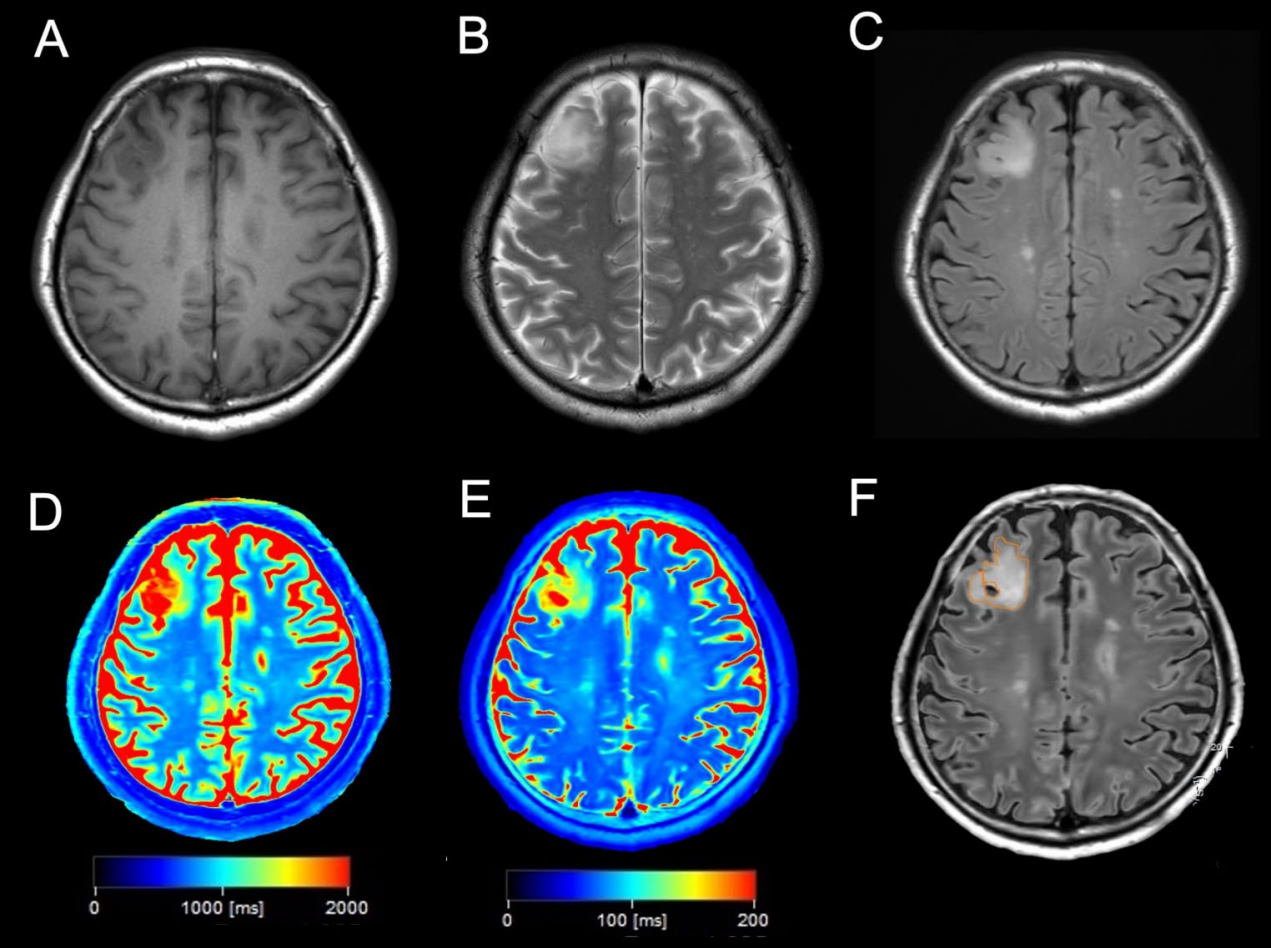
A case of a 69-year-old man with oligodendroglioma, IDH-mutant and 1p/19q-codeleted, grade 2. (**A**) T1-weighted imaging showing a heterogenous hypointense tumor in the right frontal lobe. (**B**) T2-weighted imaging showing a heterogenous hyperintense tumor in the right frontal lobe. (**C**) Fluid-attenuated inversion recovery showing a heterogenous hyperintense tumor without signal suppression in the right frontal lobe, indicating a negative T2-FLAIR mismatch. (**D**, **E**) T1 (**D**) and T2 (**E**) maps showing a heterogeneous mild prolongation of T1 and T2 values in the tumor lesion. (**F**) The ROI was drawn in tumor lesion.

Figures 2 and 3 show the representative images of patients with astrocytoma, IDH-mutant and oligodendroglioma, IDH-mutant and 1p/19q codeleted, respectively. And the additional cases were presented in supplemental Figs. 1 and 2.

## Discussion

The current study quantitatively evaluated T1, T2, and PD values for IDH-mutant diffuse glioma using synthetic MRI. Notably, our findings revealed that quantitative evaluation of diffuse glioma promoted better accuracy in differentiating astrocytoma from oligodendroglioma than did the T2-FLAIR mismatch sign. In one of the previous studies on quantitative MR analysis of diffuse glioma, Kikuchi et al. attempted to differentiate astrocytoma from oligodendroglioma by evaluating T1 and T2 relaxation times and PD value obtained from synthetic MRI [18]. They used histogram analysis of the single slice of synthetic MRI and showed that astrocytoma could be completely differentiated from oligodendroglioma using the mean, 10th percentile and 50th percentile of T2 relaxation time with cutoffs of 178 ms, 100 ms, and 148 ms, respectively. In their additional study, the combination of the 90th percentile of T1 relaxation times and the 10th percentile of T2 relaxation times showed high diagnostic performance with 94.4% sensitivity and 100% specificity [25]. In our study, the mean value of T1, T2 and PD vales of the tumor lesion from a single slice with synthetic MR showed statistically higher in astrocytomas than in oligodendrogliomas. Moreover, astrocytomas could be completely differentiated from oligodendroglioma using the mean T1 value, although T2 value alone did not completely distinguish these tumors. In the other previous studies on quantitative MR analysis with MSE T2WI and MP2RAGE analysis showed that astrocytoma with T2-FLAIR mismatch sign exhibited extremely long T1- and T2-relaxation time [11]. Consequently, higher T1 and T2 values at tumor lesion may contribute to the unique feature of the T2-FLAIR mismatch sign and to the differentiation between astrocytoma and oligodendroglioma.

The T2-FLAIR mismatch sign shows high specificity for astrocytoma, IDH-mutant, and visual qualitative assessment provides clinicians with simple and clinically useful information. One systematic review found that the T2-FLAIR mismatch sign had a sensitivity and specificity of 42% (95%CI: 34–50%), and 99% (95%CI: 96–100%) for detecting IDH-mutant, 1p/19q non-codeleted tumor, respectively [10]. However, moderate interrater agreement has sometimes been observed given the subjective nature of qualitative visual assessment and variably stringent application of defining criteria [8, 26]. Furthermore, a previous study using different MR acquisition parameters revealed that the TI for FLAIR acquisition influences the diagnostic accuracy of the T2-FLAIR mismatch sign, FLAIR scanned with TI < 2,400 ms improved the detectability of T2-FLAIR mismatch sign [12]. Despite the adjustment of the MR parameters, complete detection of astrocytoma, IDH-mutant could be difficult possibly due to the definition of T2-FLAIR mismatch sign. The original definition of T2-FLAIR mismatch sign was a complete/near-complete hyperintense signal on T2WI and relatively hypointense signal on FLAIR except for a hyperintense peripheral rim [24]. Additionally, proportion criteria of T2-FLAIR mismatch were proposed that tumors with more than 50% of T2-FLAIR mismatch lesion showed 100% specificity for identifying a 1p/19q non-codeleted tumor [27]. Some of the astrocytoma, IDH-mutant presents partial T2-FLAIR mismatch lesion, which could be a factor of the limited sensitivity of T2-FLAIR mismatch sign for the tumor [28]. In our study, ROI was simply placed at the tumor lesion in the single maximum section without considering the T2-FLAIR mismatch lesion, and the average values of T1, T2 and PD values were calculated with synthetic MRI. This technique improved the detectability of astrocytoma, IDH-mutant.

Synthetic MRI can provide T1, T2, and PD values for each pixel using only a single data acquisition and can generate image contrasts with variable T1 and T2 weighting. Although several methods for the simultaneous acquisition of T1 and T2 values through mixed sequences have been developed [29], the long acquisition times were unsuitable for clinical use. The QRAPMASTER sequence developed in 2008 has allowed clinicians to obtain multiple weighted images with a single scan at clinically applicable speeds [14]. While conventional T1- and T2-WI had difficulty in measuring T1 and T2 relaxation time, synthetic MRI with QRAPMASTER sequence can obtain T1, T2, and PD values within a short time with high reproducibility [17].

Both the T1 and T2 relaxation times have an impact on the suppression of the FLAIR signal in the T2-FLAIR mismatch sign. Given that FLAIR imaging requires longer TE and is influenced by T2 relaxation time, brain tissue on FLAIR images appears to be similar to T2WI, with higher intensity for gray matter than white matter. In addition, the FLAIR method suppresses cerebrospinal fluid signal using inversion recovery such that an increase in the T1 relaxation time of the subject decreases the degree to which longitudinal magnetization recovers, thereby showing a lower intensity in the FLAIR image [30, 31]. Therefore, quantitative evaluation of T1 and T2 values of diffuse glioma using synthetic MRI could theoretically provide better accuracy for differentiating astrocytoma from oligodendroglioma than would the visually based conventional T2-FLAIR mismatch sign.

The limitations of this study include its small sample size and retrospective design. Further studies are necessary to validate the cutoff T1 and T2 values for differentiating between molecularly diagnosed astrocytoma and oligodendroglioma.

## Conclusion

Quantitative evaluation of non-enhanced IDH-mutant diffuse glioma using synthetic MRI provided better accuracy for differentiating astrocytoma from oligodendroglioma than did the T2-FLAIR mismatch sign. Measurement of T1 and T2 values could improve the differentiation of IDH-mutant diffuse gliomas.

## Electronic supplementary material

Below is the link to the electronic supplementary material.

Supplementary Material 1

Supplementary Material 2

## Data Availability

No datasets were generated or analysed during the current study.

## Abbreviations

QRAPMASTER: Quantification of Relaxation Times and Proton Density by Multiecho acquisition of a saturation-recovery using Turbo spin-Echo Readout
FLAIR: Fluid-attenuated inversion recovery
WI: Weighted imaging
MRI: Magnetic resonance imaging
