# Supplementary figures and images for: Usefulness of synthetic MRI for differentiation of IDH-mutant diffuse gliomas and its comparison with the T2-FLAIR mismatch sign
Source: J Neurooncol. 2024 Aug 12;170(2):429–36. doi: 10.1007/s11060-024-04794-0 (PMC11538156; doi:10.1007/s11060-024-04794-0)

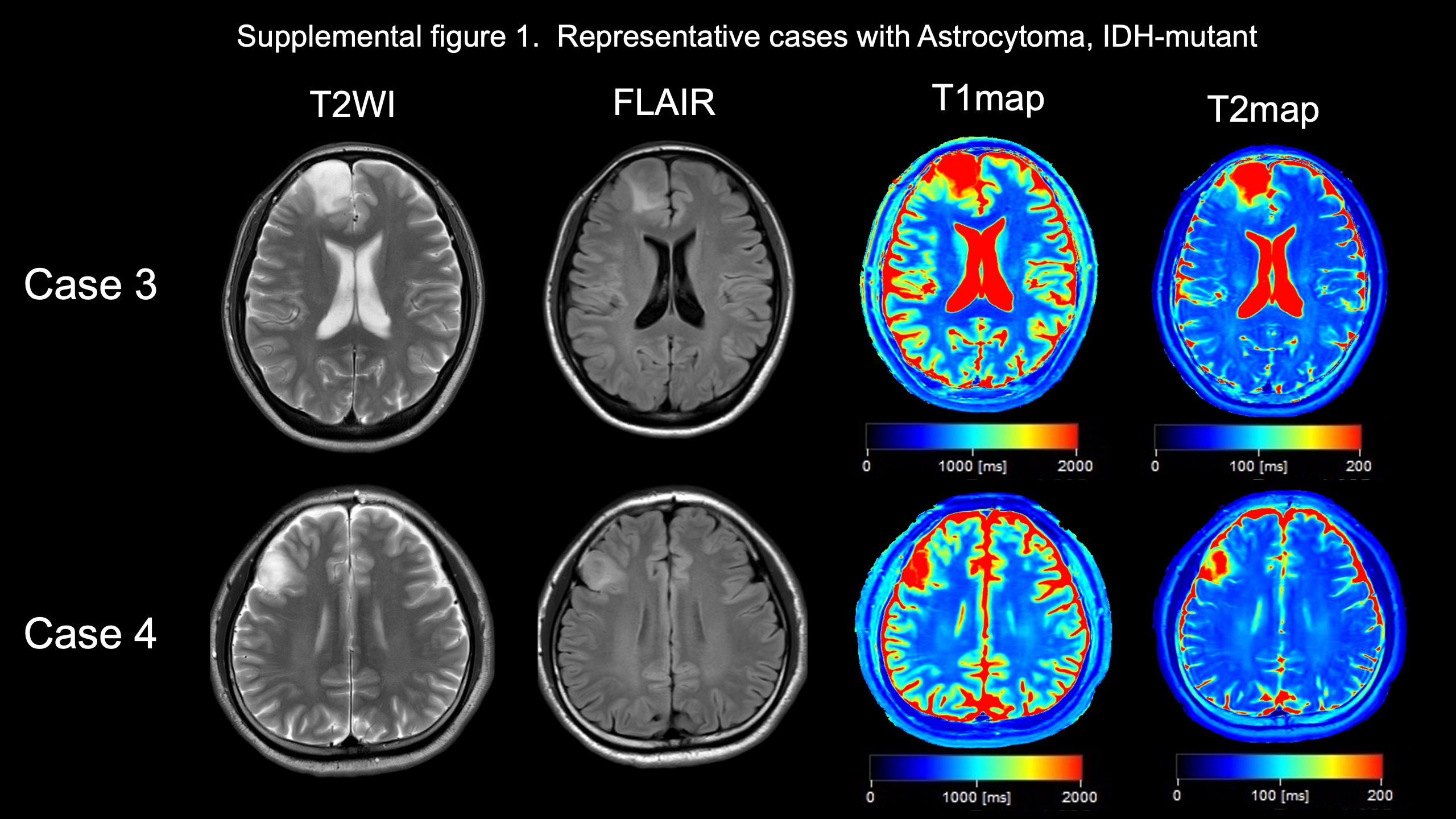

Supplement: Supplementary file 1 — Supplementary Material 1 [file 11060_2024_4794_MOESM1_ESM.jpeg]

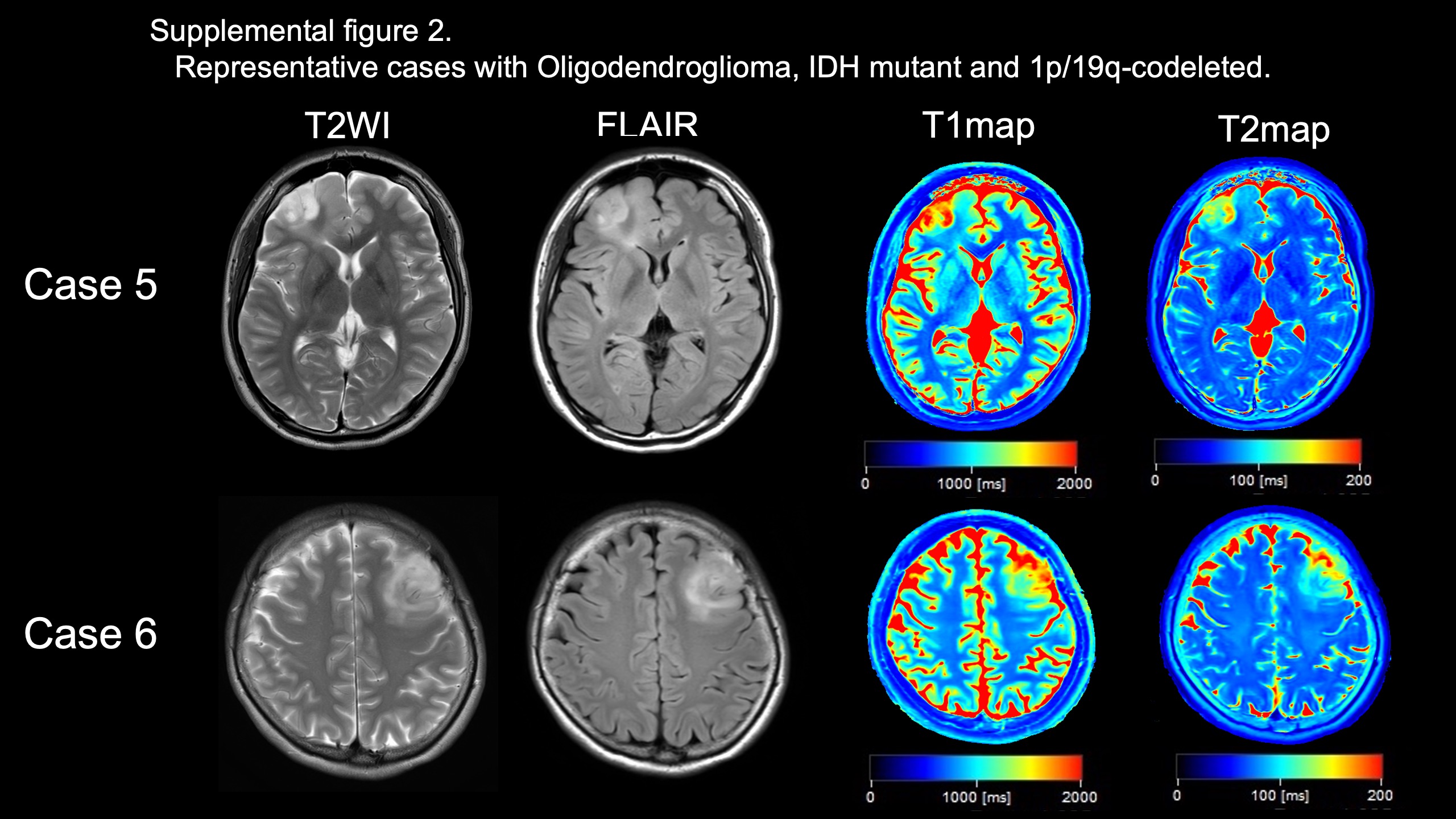

Supplement: Supplementary file 2 — Supplementary Material 2 [file 11060_2024_4794_MOESM2_ESM.jpeg]
